# High azole MICs in *Fusarium* spp.: a key factor in treatment decisions for cancer patients?

**DOI:** 10.1590/S1678-9946202567068

**Published:** 2025-10-13

**Authors:** Raquel Keiko de Luca Ito, Marcia de Souza Carvalho Melhem, Lucas Xavier Bonfietti, Milena Dropa, Juliana Possatto Fernandes Takahashi, Rogério Antonio de Oliveira, Dulcilena de Matos Castro e Silva, Maria Emília Batista de Sousa, Patrícia Rodrigues Bonazzi Pontes, Vítor Falcão de Oliveira, Afonso Rafael da Silva, João Nóbrega de Almeida, Evangelina da Motta Pacheco Alves de Araujo, Marcello Mihailenko Chaves Magri, Edson Abdala

**Affiliations:** 1 Instituto do Câncer do Estado de São Paulo, São Paulo, São Paulo, Brazil; 2 Universidade Federal do Mato Grosso do Sul, Faculdade de Medicina, Campo Grande, Mato Grosso do Sul, Brazil; 3 Universidade de São Paulo, Faculdade de Medicina, Laboratório de Investigação Médica (LIM-53), São Paulo, São Paulo, Brazil; 4 Instituto Adolfo Lutz, São Paulo, São Paulo, Brazil; 5 Universidade de São Paulo, Faculdade de Saúde Pública, São Paulo, São Paulo, Brazil; 6 Instituto Adolfo Lutz, Unidade de Patologia Quantitativa, São Paulo, São Paulo, Brazil; 7 Universidade de São Paulo, Faculdade de Medicina, Hospital das Clínicas, Divisão de Laboratório Central, São Paulo, São Paulo, Brazil; 8 Universidade de São Paulo, Faculdade de Medicina, Hospital das Clínicas, Departamento de Moléstias Infecciosas e Parasitárias, São Paulo, São Paulo, Brazil; 9 Universidade de São Paulo, Faculdade de Medicina, Instituto de Medicina Tropical de São Paulo, Laboratório de Hepatologia por Vírus (LIM-47), São Paulo, São Paulo, Brazil

**Keywords:** Antifungal susceptibility testing, Combination therapy, Fusarium, Invasive fusariosis

## Abstract

Invasive fusariosis (IF) is an exceptionally severe disease, particularly affecting patients with prolonged neutropenia. Even with antifungal therapy, mortality rates remain high, emphasizing the need for stringent control over hospital environment and further research to enhance patient outcomes. This study aims to assess the antifungal susceptibility of clinical and environmental *Fusarium* isolates and their clinical relevance in a cancer hospital. Sixteen environmental and 21 clinical isolates were identified, with the *Fusarium solani* species complex being the most common. Amphotericin B demonstrated *in vitro* activity, but most isolates exhibited elevated minimum inhibitory concentrations (MICs) for azoles. A total of 80% of patients had IF, primarily those with hematological malignancies, and the 30-day mortality rate was 43.8%. Isolates with elevated MICs were often managed with combination therapy. In conclusion, elevated azole MICs may lead to increased use of combination therapy in real-world clinical settings. Our findings emphasize the importance of careful interpretation of antifungal susceptibility testing results in IF management.

## INTRODUCTION

*Fusarium* spp. is a ubiquitous mold found in soil and water^1,2^. Initially regarded as a plant pathogen, it has increasingly been associated with opportunistic infections in humans and other animals^3^. In immunocompromised patients, *Fusarium* spp. can cause a variety of infections, ranging from localized to disseminated disease, often with high mortality rates^2^. Hospital water systems have been implicated as potential reservoirs of *Fusarium* spp. and linked to cases and outbreaks of invasive fusariosis (IF) in healthcare settings^4-6^. Additionally, most *Fusarium* species complexes exhibit elevated minimum inhibitory concentration (MIC) values to antifungals, including new broad-spectrum azoles^7,8^.

The optimal treatment strategy for patients with IF has not yet been defined. Monotherapy with voriconazole (VCZ) is frequently recommended by specialists and is in the most recent IF treatment guidelines^7,9^. Combination therapy with VCZ plus lipid formulation of amphotericin B (LAmB) is commonly used in specialized centers due to high VCZ MICs, while others prefer monotherapy.

We aimed to determine the antifungal susceptibility profiles of clinical and environmental *Fusarium* isolates and correlate them with clinical implications for patients in a cancer hospital.

## MATERIALS AND METHODS

### Ethics

The study was conducted in accordance with the Declaration of Helsinki and approved by the Ethics Committee of the Medical School of University of Sao Paulo, Brazil (protocol Nº 65155717.5.0000.0065, approved on March 8, 2017). Patient consent was waived due to the retrospective design, based on analyses of clinical and environmental samples.

### Identification of clinical and environmental Fusarium isolates

We included both clinical and environmental *Fusarium* spp. strains isolated between 2012 and 2018 at Instituto do Cancer do Estado de Sao Paulo. Clinical strains were identified using proteomic analysis (MALDI-TOF MS, VITEK, bioMérieux, USA) at the Central Laboratory Division of Hospital das Clinicas, University of Sao Paulo. Environmental *Fusarium* isolates were collected and cultured at the Mycology Laboratory of the Adolfo Lutz Institute (IAL), where phenotypic identification was performed. Both clinical and environmental isolates were preserved in the fungal culture collection at IAL and were processed for species-level identification.

### Culture revival and phenotypic characterization

Cryopreserved isolates were thawed slowly at 2–8 °C. Morphological characteristics were assessed following subculture on Sabouraud dextrose agar (SDA) with chloramphenicol at 30 °C. Pure cultures were maintained at 30 °C in SDA slants sealed with cotton plugs. Phenotypic identification included macroscopic and microscopic assessment using giant colony morphology, slide culture, and standard biochemical tests.

### MALDI-TOF MS identification

Protein-based identification was performed using MALDI-TOF MS (Bruker Daltonics, Billerica, MA, USA) with the Biotyper 3.1 database. Protein extraction followed the manufacturer’s recommended formic acid protocol. Fungal biomass was obtained from Sabouraud broth cultures incubated at 30 °C under agitation for five days. After sedimentation and washing with sterile deionized water, cell pellets were suspended in 300 µL of water and 900 µL of ethanol, vortexed, and centrifuged at 15,000 rpm. After ethanol evaporation, 50 µL of 70% formic acid and 50 µL of acetonitrile were added. The extract was vortexed, centrifuged, and 1 µL of supernatant was mixed with 1 µL of α-cyano-4-hydroxycinnamic acid (CHCA) matrix and applied to the target plate. Identification was interpreted based on log(score) values according to the manufacturer’s guidelines^10,11^.

### DNA extraction, ITS, and TEF1α sequencing

Genomic DNA was extracted using a modified cetyltrimethylammonium bromide (CTAB) method. The internal transcribed spacer (ITS) region of the rDNA was amplified using ITS1 (5'-TCCGTAGGTGAACCTGCGG-3') and ITS4 (5'-TCCTCCGCTTATTGATATGC-3') primers^12^, under the following cycling conditions: 94 °C for 3 min; 30 cycles of 94 °C for 30 s, annealing at 55 °C for 30 s, 72 °C for 1 min; and a final extension at 10 °C. Amplicons were purified using the GFX PCR DNA and Gel Band Purification Kit (GE Healthcare, UK) or enzymatically with Exonuclease I and FastAP alkaline phosphatase (Thermo Fisher Scientific, USA). Sequencing reactions were performed with ITS1 and ITS4 primers under the following conditions: 95 °C for 1 min; 28 cycles of 95 °C for 15 s, annealing at 50 °C for 45 s, and 60 °C for 4 min. Polymerase chain reactions (PCR) targeting the translation elongation factor 1-alpha ( TEF1α) gene were performed using PCR Master Mix (Promega, USA) in a thermal cycler (Applied Biosystems, USA) with EF1 (5'-ATGGGTAAGGARGACAAGAC-3') and EF2 (5' GGARGTACCAGTSATCATG-3') primers^13^. The thermal cycling protocol included an initial denaturation at 94 °C for 90 s, followed by 40 denaturation cycles at 94 °C for 30 s, annealing at 55 °C for 90 s, and extension at 68 °C for 2 min, with a final extension at 68 °C for 5 min. PCR products were visualized by electrophoresis on a 2% agarose gel at 100 V for 30 min. For enhanced resolution and species-level identification, TEF1α gene sequencing was conducted using EF1 and EF2 primers^14-16^. Purification followed a modified protocol from the DYEnamic^™^ ET Dye Terminator Kit (GE Healthcare, UK). After drying, pellets were resuspended in 3 µL of formamide, vortexed, centrifuged, and analyzed on an ABI 3130 Genetic Analyzer (Applied Biosystems). Sequences were aligned and edited using BioEdit (version 7.0.5.3, Hall) and compared to reference sequences using BLASTn (National Center for Biotechnology Information, USA). Species identification was assigned when sequence identity was ≥ 99% with reference sequences.

### Antifungal susceptibility testing (AST)

AST followed the Clinical and Laboratory Standards Institute (CLSI) guidelines (M38//M61)^17,18^. MICs were determined as the lowest drug concentration that completely inhibited fungal growth. Because CLSI clinical breakpoints—susceptible, intermediate, resistant—are not available for *Fusarium* spp., we interpreted MICs using epidemiological cutoff values (ECVs) when available^8,18^. Isolates were classified as non-wild type (NWT) when MICs exceeded the following thresholds: AmB > 8 mg/L (*F. solani* species complex [FSSC] and *F. oxysporum* species complex [FOSC]); VCZ > 16 mg/L (FOSC) or > 32 mg/L (FSSC); posaconazole (PCZ) > 8 mg/L (FOSC) or > 32 mg/L (FSSC); and itraconazole (ITZ) > 32 mg/L (FSSC and FOSC)^8^. ECVs for isavuconazole (ISA) in *Fusarium* spp. have not been established. In this context, “elevated MICs” refers to values above these ECVs, indicating a NWT phenotype.

Epidemiological and clinical data were retrospectively collected from electronic medical records. This study was approved by the local ethics committee, and informed consent was waived. We adhered to the traditional *Fusarium* spp. nomenclature based on global consensus guidelines^19^. Colonization was defined as the isolation of *Fusarium* spp. without microbiological, clinical, or radiological evidence of active infection. Proven invasive fungal disease was diagnosed based on the EORTC/MSG 2020 criteria^20^.

## RESULTS

*Fusarium* strains were isolated from 16 hospital environmental samples (five from water and 11 from air) and 21 clinical samples from 20 patients, including blood (47.6%; 10/21), skin biopsy (19.05%; 4/21), urine (19.05%; 4/21), nasal mucosa (9.5%; 2/21), and pleural effusion (4.8%; 1/21). Species identification and MICs for clinical and environmental isolates are detailed in Table 1.

**Table 1:** Characterization of clinical and environmental *Fusarium* spp. isolates.

		a) Species identification and MICs of *Fusarium* spp. isolates from cancer patients									
N	Site	Species complex	Cryptic species	MIC (mg/L)					Clinical presentation	Treatment	Outcome (30 days)
				AMB	TBF	VCZ	PCZ	ISA			
1*	Blood	FSSC	*F. keratoplasticum*	2	>8	>32	>8	>0.5	Fungemia	LAmB+VCZ	Died
2*	Blood	FSSC	*F. solani*	2	8	>32	>8	>0.5			
*3*	Blood	FSSC	*F. solani*	1	64	>32	>8	>0.5	Disseminated	LAmB	Died
*4*	Blood	FSSC	*F. solani*	2	0,5	>32	>8	ND	Disseminated	LAmB+VCZ	Died
*5*	Skin	FSSC	*F. petroliphilum*	2	4	>32	>8	>0.5	Disseminated	LAmB+VCZ	Survived
*6*	Blood	FSSC	*F. solani*	1	4	>32	>8	>0.5	Disseminated	LAmB+VCZ	Survived
*7*	Urine	FOSC	*F. oxysporum* f. sp*. ciceris*	2	2	>32	>8	>0.5	Colonized	None	Survived
*8*	Urine	FSSC	*F. petrophilum*	0.5	ND	>32	>8	>0.5	Colonized	None	Survived
*9*	Urine	FOSC	No signif similar	1	8	>32	>8	>0.5	Colonized	None	Survived
*10*	Urine	FSSC	*F. solani*	0.5	4	>32	>8	>0.5	Colonized	None	Died
*11*	Blood	FSSC	*F. solani*	2	2	>32	>8	>0.5	Disseminated	LAmB	Died
*12*	Blood	FSSC	*F. keratoplasticum*	1	>8	>32	>8	>0.5	Disseminated	LAmB+VCZ	Survived
*13*	Nasal mucosa	FSSC	*F. solani*	1	4	>32	>8	>0.5	Sinopulmonary	LAmB	Survived
*14*	Skin	FSSC	*F.keratoplasticum*	1	2	>32	8	>0.5	Localized	LAmB+VCZ	Survived
*15*	Blood	FSSC	*F. solani*	2	4	>32	>8	>0.5	Fungemia	LAmB+VCZ	Died
*16*	Blood (+ skin)	FSSC	*F.vanetteni*	1	ND	>8	ND	>0.5	Disseminated	LAmB+VCZ	Survived
*17*	Blood	FSSC	*F.vanetteni*	1	>8	4	>8	>0.5	Fungemia	None	Died
*18*	Skin	FSSC	*F. petrophilum*	2	>8	>32	>8	>0.5	Disseminated	LAmB+VCZ	Survived
*19*	Nasal mucosa	FSSC	*F.vanetteni*	4	>8	4	2	>0.5	Disseminated	LAmB+VCZ	Survived
*20*	Pleural fluid	FSSC	*F.vanetteni*	1	ND	>8	ND	>0.5	Pulmonary	None	Died
*21*	Skin	Fusarium sp.	Unknown	1	ND	>32	>8	>0.5	Disseminated	LAmB+VCZ	Died
		**b) Species identification and MICs of *Fusarium* isolates from hospital environment**									
*22*	water	FSSC	*F. solani*	2	4	2	>8	ND	-	-	
*23*	air	*Fusarium* sp.	Unknown	2	ND	>32	0.03	0.03	-	-	
*24*	water	FOSC	*F.verticilloides*	>8	1	>32	>8	>0.5	-	-	-
*25*	water	FOSC	*F. oxysporum* f. sp*. ciceris*	>8	1	>32	>8	>0.5	-	-	-
*26*	water	FSSC	*F.petrophilum*	1	1	>32	1	>0.5	-	-	-
*27*	water	FSSC	*F.petrophilum*	1	1	>32	>8	>0.5	-	-	-
*28*	air	FSSC	*F.petrophilum*	2	1	>32	>8	>0.5	-	-	-
*29*	air	FSSC	*F.petrophilum*	4	1	>32	>8	>0.5	-	-	-
*30*	air	FOSC	*F. oxysporum* f. sp. *ciceris*	2	>8	>32	>8	>0.5	-	-	-
*31*	air	*Fusarium* sp.	Unknown	1	2	>32	>8	>0.5	-	-	-
*32*	air	FSSC	*F.petrophilum*	2	>8	>32	>8	>0.5	-	-	-
*33*	air	FSSC	*F.petrophilum*	0.5	1	>32	>8	>0.5	-	-	-
*34*	air	FOSC	Unknown	2	>8	>32	>8	>0.5	-	-	-
*35*	air	FOSC	*F. oxysporum* f. sp*. ciceris*	1	0.5	>32	1	>0.5	-	-	-
*36*	air	*Fusarium* sp.	Unknown	1	0.25	4	>8	>0.5	-	-	-
*37*	air	*Fusarium* sp.	Unknown	0.25	0.25	2	>8	>0.5	-	-	-

*Isolates 1 and 2 were from the same patient; AmB = Amphotericin; ICZ = Itraconazole; VCZ = Voriconazole; PCZ = Posaconazole; ISA = Isavuconazole; LAmB = Lipid formulation of AmB; FSSC = *Fusarium solani* species complex; FOSC = *Fusarium oxysporum* species complex; ND = Not determined; NA = Not available.

FSSC was the most frequently isolated group from both clinical (85.7%; 18/21) and environmental samples (43.8%; 7/16). Among clinical isolates, *F. solani* was the most commonly identified cryptic species (38.1%; 8/21), whereas *F. petroliphilum* predominated in environmental samples (37.5%; 6/16).

AmB showed *in vitro* activity for all clinical and most environmental isolates (only 2/16 isolates presented MIC above 8 mg/L). Most *Fusarium* spp. isolates were classified as NWT to azoles. Specifically, 19/21 (90.5%) clinical and 13/16 (81.3%) environmental isolates had MICs > 32 mg/L for ITZ; 17/21 (81.0%) clinical and 13/16 (81.3%) environmental isolates had MICs > 32 mg/L for VCZ; and 18/19 (94.7%) clinical and 13/16 (81.3%) environmental isolates tested for PCZ had MICs > 8 mg/L. Although ECVs for ISA against *Fusarium* species complexes have not yet been established, all 20 clinical isolates and 14/15 (93.3%) environmental isolates exhibited MICs > 0.5 mg/L (Table 2).

**Table 2:** Antifungal susceptibility testing results for clinical and environmental *Fusarium* spp. isolates.

Antifungal	Clinical isolates with MIC above threshold n/N (%)	Environmental isolates with MIC above threshold n/N (%)	Threshold (mg/L)*
Amphotericin B	0/21 (0%)	2/16 (12.5%)	>8
Itraconazole	19/21 (90.5%)	13/16 (81.3%)	>32
Voriconazole	17/21 (81.0%)	13/16 (81.3%)	>32
Posaconazole**	18/19 (94.7%)	13/16 (81.3%)	>8
Isavuconazole***	20/20 (100%)	14/15 (93.3%)	>0.5

*Thresholds based on established epidemiological cutoff values (ECVs), when available; **Two clinical isolates not tested for PCZ; ***One clinical and one environmental isolate were not tested for ISA. ECVs for ISA against *Fusarium* spp. are not yet established; threshold shown for descriptive purposes only.

Sixteen out of 20 patients (80%) had *Fusarium* spp. isolated from sterile sites and/or presented signs and symptoms suggestive of IF and were classified as infected. The remaining four patients were considered colonized. Hematological malignancies were the most common underlying condition among infected patients (75%, 12/16), whereas all colonized patients had solid tumors.

Most IF cases (68.8%; 11/16) were treated with combination therapy using a LAmB plus VCZ, with seven patients surviving (63.6%). Three cases received only a LAmB, with one surviving (33.3%). No patient received VCZ monotherapy. Two patients died before the infection was diagnosed and did not receive antifungal therapy. The 30-day mortality rates for infected and colonized patients were 43.8% and 25.0%, respectively.

## DISCUSSION

In this study, we observed that most environmental and clinical *Fusarium* spp. isolates exhibited elevated MICs for triazoles. This susceptibility pattern may have future clinical implications for IF management, potentially leading to increased use of combination therapy with AmB and VCZ, and the absence of VCZ monotherapy.

Most studies evaluating *in vitro* antifungal resistance of *Fusarium* spp. isolates also found elevated MICs for azoles. Espinel-Ingroff *et al*.^8^ established ECVs for *Fusarium* spp. based on the CLSI broth dilution method for AST. MICs were higher for azoles compared to AmB, with FSSC isolates presenting higher MICs for VCZ and PCZ compared to FOSC isolates.

Santos *et al*.^21^ evaluated the *in vitro* activity of seven antifungal drugs against 98 *Fusarium* isolates recovered from fungal keratitis patients, with AmB showing the highest activity.

Pfaller *et al*.^22^ evaluated the activity of ISA against 24 *Fusarium* strains, 18 of which were *F. solani*. All 24 isolates had MICs above 4 µg/mL. Broutin *et al*.^23^ assessed the *in vitro* susceptibility of 75 *Fusarium* isolates to ISA and found that most strains had MICs above 16 µg/mL, with no significant differences between species complexes. The predominant species were *F. proliferatum*, FOSC, and *F. petroliphilum*. Further studies are needed to establish ISA ECVs for *Fusarium* spp. Marinelli *et al*.^24^ conducted a systematic review of studies published from 2011 to 2021 on major fungal pathogens, including *Fusarium* species. Of the 315 articles initially identified, 20 were selected, comprising 137 *Fusarium* strains. The findings revealed median MIC values of ≥ 16 mg/L for ICZ and ISA, whereas VCZ exhibited median MIC values of 2–8 mg/L for non-FSSC isolates.

The largest study assessing AST in *Fusarium* spp. included 128 isolates, distributed across three species complexes: FOSC, FFSC, and FSSC. The MICs of AmB, VCZ, PZC, ISA, and terbinafine (TBF) were determined. AmB demonstrated the best i*n vitro* activity for *Fusarium* spp., whereas FSSC exhibited the lowest *in vitro* susceptibility to antifungals, including ISA^25^.

Therefore, the clinical significance of this *in vitro* antifungal resistance remains unclear due to the lack of clinical trials in patients with IF^7,26^. Available data are limited to case series involving small cohorts. Two studies by Nucci *et al*.^26,27^ found no correlation between MIC values and clinical outcomes in patients with hematologic diseases and IF, suggesting that AST should be primarily used for epidemiological purposes. Given the absence of CLSI or EUCAST clinical breakpoints for *Fusarium* spp., MIC values must be interpreted with caution, since exceeding the ECV does not necessarily indicate therapeutic failure but may suggest a reduced probability of clinical success, particularly in invasive disease. Moreover, the consistent detection of elevated MICs in environmental isolates may support the hypothesis of a potential environmental reservoir contributing to difficult-to-treat infections. In our series, FSSC was retrieved from both patients and the hospital environment, with similar susceptibility profiles, suggesting an environmental role in nosocomial acquisition. Further clinical studies, particularly involving larger population , including other immunocompromised groups such as patients with solid tumors, are warranted to assess the clinical impact of probable resistance in IF management.

In our study, awareness of AST results may have contributed to the increased use of combination therapy. To date, no randomized trials have evaluated the efficacy of antifungal drugs for IF treatment. The largest multicenter retrospective study (236 patients, 1985–2011, 44 centers, 11 countries) analyzed 206 patients receiving monotherapy with DAmB (110 patients), VCZ (38 patients), and LAmB (34 patients). The 90-day survival rates were 27% for DAmB, 53% for VCZ, and 48% for LAmB^26^. Other studies have also reported primary treatment with VCZ, LAmB, and DAmB. Few case reports have documented successful outcomes with ISA, TBF, or PCZ, while no response was observed with echinocandin therapy^7^.

Current global guidelines strongly recommend VCZ or LAmB as primary treatment for IF, discouraging the use of DAmB if other active antifungal options are available. Other agents are only marginally recommended. Combination therapy is often used as primary treatment due to disease severity, challenges in achieving adequate VCZ trough levels, and high MICs for azoles and polyenes. Early step-down to monotherapy, guided by MIC results, is also recommended^7^.

Our findings align with and complement those reported by Hino *et al*.^28^, who investigated IF epidemiology in patients with hematologic malignancies and identified hospital drain outlets as relevant environmental reservoirs for *Fusarium* spp., particularly *F. petroliphilum* and other members of the FSSC. In their study, *Fusarium* DNA and viable cultures were predominantly retrieved from drain outlets, and droplet-mediated dispersion was demonstrated experimentally, highlighting an overlooked transmission route. Similarly, in our study, a substantial proportion of environmental isolates were retreived from water and air samples in hospital wards previously occupied by patients with IF, with a predominance of FSSC, including *F. petroliphilum*. The comparable species distribution and elevated azole MIC profiles observed in both environmental and clinical isolates suggest a potential epidemiological link, reinforcing the hypothesis that hospitals may contribute significantly to IF acquisition. These findings underscore the importance of environmental surveillance and preventive strategies targeting water-related infrastructure to mitigate the risk of nosocomial IF in high-risk patients.

Limitations of this study include its single-center retrospective design and the small number of isolates recovered from both patients and the environment. The limited sample size rendered comparative analyses and MIC-outcome correlations underpowered and not robust; therefore, only descriptive results are presented. Furthermore, because high-resolution genotyping (e.g., multilocus sequence typing, microsatellite typing, or whole-genome sequencing) was not performed, clonal relatedness and potential transmission between environmental and clinical *Fusarium* isolates cannot be established. Consequently, the observed concordance in species composition and azole MIC distributions should be considered hypothesis-generating and is insufficient to confirm a definitive reservoir. Additionally, *Fusarium* species complex identification and antifungal susceptibility testing were not available for all isolates. Despite these limitations, the detection of elevated MICs in both environmental and clinical isolates may encourage clinicians to consider combination antifungal therapy for empirical and targeted management of IF.

## CONCLUSION

Our study demonstrated that most clinical and environmental *Fusarium* spp. isolates exhibited elevated azole MICs, including newer agents. Although the clinical impact of this high MIC pattern has not been fully established, preventive measures should be implemented in hospital settings to protect high-risk patients from environmental exposure to *Fusarium* spp. The outbreak investigation context provided a unique opportunity to assess the prevalence and distribution of elevated azole MICs among both clinical and environmental isolates. Furthermore, AST of *Fusarium* spp. may have important clinical implications, particularly in guiding the selection of targeted therapy for IF.

## Data Availability

The anonymized dataset generated during this study is available from the corresponding author upon reasonable request.
